# Supplementary material for: Assessing Barriers Encountered by Women in Cervical Cancer Screening and Follow-Up Care in Urban Bolivia, Cochabamba
Source: Healthcare (Basel). 2022 Aug 24;10(9):1604. doi: 10.3390/healthcare10091604 (PMC9498362; doi:10.3390/healthcare10091604)
Supplement: Supplementary file 1 [file healthcare-10-01604-s001.zip › Supplementary Material File S1.pdf]

Figure S1: Covered municipalities including delimitation of provinces in the department of Cochabamba

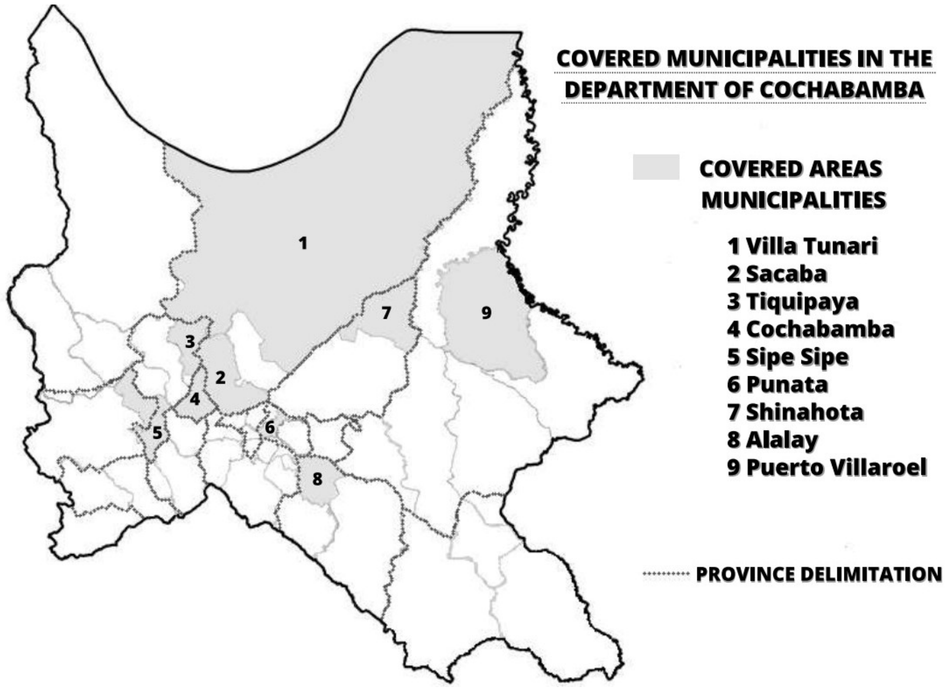

Table S1: Covered geographical areas

**Municipality**	**Province**
Puerto Villarroel	Carrasco
Cochabamba	Cercado
Sacaba	Chapare
Villa Tunari	Chapare
Alalay	Mizque
Punata	Punata
Sipe Sipe	Quillacollo
Tiquipaya	Quillacollo
Shinahota	Tiraque

Table S2: Interviewed healthcare professionals

**Subject**	**Profession**	**Geographical area**	**Participation in the pilot project**	**Municipality**
HCP#1	Doctor gynecologist	Hospital Viedma	Yes	Cochabamba
HCP#2	Doctor generalist	Hospital Chipiriri (Tropico)	yes	Villa Tunari
HCP#3	Doctor Gynecologist	Hospital Ibuelo (Tropico)	yes	Villa Tunari
HCP#4[#]	Nurse	Hospital Villa Tunari (Tropico)	yes	Villa Tunari
HCP#5	Nurse	Hospital Chipiriri (Tropico)	yes	Villa Tunari
HCP#6	Chemist	Pucarita	yes	Cochabamba
HCP#7	Nurse	Villa Tunari (Tropico)	No*	Villa Tunari

HCP#8	Doctor generalist	Chipiriri (Tropico)	Yes	Villa Tunari
HCP#9	Doctor gynecologist	Cercado	Yes	Cochabamba
HCP#10	Nurse	Cercado	Yes	Cochabamba

*She collaborated, #Health provider and patient, she completed both questionnaires

Table S3: Interviewed female patients

Subject	occupation	Geographical area where the interview take place	Municipality
Woman#1	Merchantwoman	Punata	Punata
Woman#2	/	Sipe Sipe	Sipe Sipe
Woman#3	Merchantwoman	Punata	Punata
Woman#4	Merchantwoman	Interviewed at the health centrum of Sipe Sipe but lived in Parotani	Sipe Sipe
Woman#5	Dressmaker	Cercado	Cochabamba
Woman#6	Dentist	Cercado - interviewed by phone	Cochabamba
Woman#7	/	Villa Tunari (Tropico) - interviewed by phone	Villa Tunari
Woman#8	/	Used to live in Tropico moved to Chili - interviewed by phone	Villa Tunari
Woman#9	Unemployed	Ivirgarzama - interviewed by phone	Puerto Villaroel
Woman#10	/	Alalay -interviewed by phone	Alalay
Woman#11	Housewife	Shinaota -interviewed by Phone	Shinaota
Woman#12	Merchantwoman	Punata	Punata
Woman#13	Orthodontist	Punata -interviewed by phone	Punata
Woman#14	HCP	Cercado	Cochabamba
Woman#15	Housewife	Punata	Punata

Table S4: Interviewed health promoters/promotoras

Subject	Occupation	Geographical area where the interview take place	Municipality
Promotora#1	Self-employed	Sipe Sipe	Sipe Sipe
Promotora#2	/	Cercado	Cochabamba
Promotora#3	Accountant	Punata	Cochabamba
Promotora#4	Community leader	Sipe Sipe	Sipe Sipe

Table S5: Interviewed medical care settings chosen by women

	**% General Check-up (N=244)**	**% Gynecological Check-up (N=244)**
Public hospital	32.38	26.23
Caja de salud	4.1	2.05
General practitioner	11.46	0
Health centers	15.98	14.75
Private hospital	24.59	27.05
Traditional practitioner	0.41	0
Follow-up within pilot project	0	0.41
No check-up	11.07	18.03

Table S6: Means of announcing the results

	**HCPs (%, N=36)**	**Women (%, N=48)**
Phone calls	19.44	14.58
Letters	0	0
Via general practitioner	27.77	27.08
Via gynecologist	19.44	31.25
Via a promotora (pilot project)	0	8.33
When patient comes for a consultation	50	0
At the patient's home	5.56	0
No results were received	0	10.42
Via medical laboratory	0	8.33
Direct personal contact	8.33	0

* Respondents could select multiple choices.
