# Supplementary material for: Assessing Barriers Encountered by Women in Cervical Cancer Screening and Follow-Up Care in Urban Bolivia, Cochabamba
Source: Healthcare (Basel). 2022 Aug 24;10(9):1604. doi: 10.3390/healthcare10091604 (PMC9498362; doi:10.3390/healthcare10091604)
Supplement: Supplementary file 1 [file healthcare-10-01604-s001.zip › Supplementary Material File S2.pdf]

Guia de entrevista para mujeres VPH+

Introducción:

Buenos días, en el marco de un estudio realizado en colaboración entre la Universidad Libre de Bruselas y la Universidad Mayor San Simón, estamos interesados en la detección y el control del VPH de las mujeres diagnosticadas con una infección.

Todo lo que será discutido aquí es completamente confidencial y anónimo (datos anonimizados)
La entrevista dura aproximadamente de 30 minutos. ¿Aceptaría participar? ¿Podríamos preguntarle también si no tiene problema con el hecho que grabemos la entrevista? → la grabación será únicamente utilizada para nuestro análisis y será destruida luego de esto.

Sepa por favor que puede finalizar esta entrevista en cualquier momento y también que puede no responder algunas preguntas que puedan hacerle sentir incómodo/a.

Preguntas demográficas	1.Cuál es su nombre?<br>2.Cuántos años tiene?<br>3.Dónde vive? en esta comunidad o en otra?<br>4.Cuantos hijos tiene? cual es la edad del mayor y del menor?<br>5.Cuál es su estado civil?<br>6.Ha que se dedica usted?<br>7.En qué lugar hace sus consultas médicas?a qué distancia se encuentra? ¿No hay otro más cercano?<br>8.En su último embarazo qué pruebas le realizaron?
Caracterizar la información que conocen las mujeres	1.¿Qué sabe usted sobre el virus de papiloma humano? sobre su transmisión y riesgos?<br>2.¿Cómo obtuvo usted esa información?<br>3.¿De esa información que usted tiene del VPH que le es difícil entender?<br>4.Recibió alguna explicación sobre el tratamiento que debe seguir<br>5.Hay algo que no entendió sobre la explicación y/o tiene alguna duda sobre la infección o tratamiento a seguir?<br>6.Cuáles cree usted que son las consecuencias si no sigue el tratamiento<br>7.Qué es lo que más le preocupa o asusta del tratamiento que tiene que seguir?
Observar cómo se realiza el anuncio del diagnóstico	1.En los últimos 3 años asistió usted al ginecólogo? cuantas veces?<br>Y cuando fue su último PAP?(Le realizaron otras pruebas, VIH, otras)<br>2 Usted se realizo una auto toma, que le parece este procedimiento?<br>3.Qué pruebas se realizó? Pap/hiv/Auto toma y cuando fue la última vez<br>4.Ha recibido usted los resultados de VPH

	5.Cuánto tiempo demoraron los resultados, cómo recibió? y Quien se los entregó?<br>6.Qué información se le proporcionó cuando recibió los resultados? como?<br>7.Le han dicho que pasos debe usted seguir ó qué debe hacer<br>8.Seguiría usted esos pasos ?<br>9.Ha programado una cita después de recibir los resultados<br>10.Si es SI ¿como lo hizo? si es NO ¿que le impidió hacerlo?<br>11.Que tendría que pasar para que usted vaya al médico para hacer su tratamiento<br>12.Estaría de acuerdo que el laboratorio le comunique por teléfono los resultados.
Identificar problemas, dificultades y aciertos para la atención de salud de las mujeres (empatia/apatia/interés o no? entorno social, aspectos culturales)	1.En caso de una urgencia de salud tiene alguna dificultad para conseguir atención médica?<br>2. Cuales son sus principales dificultades para asistir al Médico/ginecólogo? (Tiempo, dinero, transporten trabajo, ayuda con los hijos)<br>3.Conoce usted el costo del tratamiento, le ha faltado dinero para pagar analisis/medicinas que le pidio el medico<br>4.En el último año ha ido mas veces al medico por la salud de su familia o por la de usted?
Opinión del entorno social y pareja	-Ha hablando de sus resultados con algunos familiares o con amigas? Si es si, que le han dicho? si es no, le preocupa que sus familiares y amigas se enteren de sus resultados?<br>-EL pensamiento (de familiares y amig@s)importa para que usted siga el tratamiento le hace cambiar su actitud de seguir el tratamiento y volver a ver al médico?<br>-Eso le impide ir al médico ?<br>-Ha hablado de su resultado HPV+ con su pareja?<br>si es si, que piensa el al respecto?le ha traido alguna dificultad<br>saber el resultado (HPV+) ha cambiado la relación con su pareja?
medicina tradicional y religion	1.ha buscado alguna vez a un curandero, yatiri, partera, médico tradicional? y por un tratamiento para el virus? Pq?<br>si es si; confía usted en el/ella?<br>2.Cree que él/ ella podría curarle de una infección al HPV+<br>3.El médico tradicional le recomendo algun tratamiento? cual?<br>4.Si el curandero, yatiri, partera, médico tradicional estuviera con el ginecólogo en la consulta usted haría el tratamiento?<br>6.Asiste usted a alguna iglesia? desde cuando?<br>7.Cree usted que su fé le podría sanar de una infección de HPV?<br>8 hay algo que le gustaría agregar?

Guia de entrevista para médicos, promotoras de salud y otros profesionales

Introduccion:

Buenos días, en el marco de un estudio realizado en colaboración entre la Universidad Libre de Bruselas y la Universidad Mayor San Simón, estamos interesados en la detección y el control del VPH de las mujeres diagnosticadas con una infección.

Todo lo que será discutido aquí es completamente confidencial y anónimo (datos anonimizados)
La entrevista dura aproximadamente de 30 minutos. ¿Aceptaría participar? ¿Podríamos preguntarle también si no tiene problema con el hecho que grabemos la entrevista? → la grabación será únicamente utilizada para nuestro análisis y será destruida luego de esto.

Sepa por favor que puede finalizar esta entrevista en cualquier momento y también que puede no responder algunas preguntas que puedan hacerle sentir incómodo/a.

**objetivos**	**preguntas**
observar cómo se realiza el anuncio del diagnóstico	1. Cual es su profesión/cargo en el proyecto?<br>2. hace cuanto tiempo ejerce su profesión/cargo.<br>3. Puede usted relatar la manera como se entregan los resultados HPV+ a las mujeres?<br>4. Qué consideraciones precauciones y otros se tiene para entregar los resultados HPV+<br>5. Existen estándares de procedimiento? de explicación del resultado?<br>6. Luego de la entrega de resultados que pasos debe seguir la paciente?
caracterizar la información que conocen las mujeres sobre VPH+	1. sabe usted dónde se informan las mujeres sobre el VPH?<br>2. en el centro de salud o la consulta se da información sobre VPH a las mujeres (publico en general) (si es si)<br>3. cuales son las principales ideas que se transmite a las mujeres sobre el VPH?<br>4. se dan consejos de prevención de infecciones de transmisión sexual à las mujeres? cuales?<br>5. Se ha realizado usted la autotoma ? qué sensación le produjo aprender los resultados ?<br>6. Qué es lo que usted piensa en su calidad de promotora y de mujer sobre la infección ?

Tratamiento	1. Como se explica a las mujeres el tratamiento a seguir (ideas claves para explicar el tratamiento a seguir)<br>2. de lo que usted ha observado, que es lo que mas les cuesta comprender a las mujeres sobre el tratamiento?<br>3. que ha escuchado usted de las mujeres sobre sus resultados (VPH+, reacciones, consideraciones, preguntas, preocupaciones y otros)
identificar problemas, dificultades, aciertos en la rélacion profesionales de salud con las mujeres (empatia/ apatia/ intéres o no)	1. según su opinión, cuáles son las razones por las cuales las mujeres que han sido diagnosticadas con el VPH+ no vienen al seguimiento?<br>2. que razones de orden personal u otras indican las mujeres para no asistir a su tratamiento?<br>3. que tienen en común las mujeres que no vuelven a su seguimiento médico?<br>4. que tienen en común las mujeres que realizan su seguimiento y tratamiento médico?<br>5. en su experiencia, qué estrategias utilizadas le ha dado mejor resultado para convencer a las mujeres de seguir su tratamiento médico?<br>6. que cosas cree que necesita para hacer más eficiente este trabajo?
medicina tradicional (curandero, partera, yatiri, jampiri)	1. sabe usted si las mujeres acuden más a los practicantes de “medicina tradicional” que a los servicios de salud?<br>2. ha escuchado o conoce de algun tratamiento que hubiera implementado la “medicina tradicional” para esta infección VPH?<br>3. ha tenido alguna experiencia de trabajar colaborativamente con practicantes de medicina tradicional?<br>4. si se presentara la oportunidad estaría dispuesto a trabajar con ellos colaborativamente?<br>5. y que de alguna iglesia?
