# Supplementary material for: Assessing Barriers Encountered by Women in Cervical Cancer Screening and Follow-Up Care in Urban Bolivia, Cochabamba
Source: Healthcare (Basel). 2022 Aug 24;10(9):1604. doi: 10.3390/healthcare10091604 (PMC9498362; doi:10.3390/healthcare10091604)
Supplement: Supplementary file 1 [file healthcare-10-01604-s001.zip › Supplementary Material File S3.pdf]

CUESTIONARIO DE INVESTIGACIÓN DESTINADO A MUJERES

En el marco de un estudio realizado en cooperación entre la Universidad Libre de Bruselas y la Universidad Mayor de San Simon, estamos realizando un estudio,con el objetivo de aumentar los conocimientos sobre la experiencia que tienen las mujeres en torno al Virus del Papiloma Humano. Los resultados obtenidos en este estudio serviran a mejorar la comunicación entre las pacientes y el personal de salud, de mismo que el seguimiento propuesto por el personal de salud a las pacientes.

Todas las informaciones dadas en este cuestionario serán completamente anónimas, confidenciales y utilizadas únicamente en el marco de nuestra investigación universitaria.

1. ¿Cuál es su edad?

- ○ - Entre 15-20 años
- ○ - Entre 21-35 años
- ○ - Entre 36-50 años
- ○ - Mas de 50 años

2. ¿Qué tipo de estudios ha hecho?

- ○ - Primaria/basico
- ○ - No termine la primaria/basico
- ○ - Secundaria/bachiller
- ○ - No termine la segundaria/bachiller
- ○ - Formacion tecnica
- ○ - Universidad
- ○ - Ninguno

3. ¿Donde hace usted su chequeo/control médico generalmente?

- ○ - Hospital publico
- ○ - Hospital privado
- ○ - Centros de salud
- ○ - Caja de salud
- ○ Otro (precisar)
- ○ - Médicos particulares
- ○ - Practicantes tradicionales (curanderos, yatiris, naturista, brujo…)
- ○ - No hago ningún chequeo/control

4. ¿Donde hace usted su chequeo/control ginecológico?

- ◯ - Hospital publico
- ◯ - Hospital privado
- ◯ - Centros de salud
- ◯ - Caja de salud
- ◯ - Médicos particulares o mi medico
- ◯ - Hago mi seguimiento con el ginecólogo del proyecto
- ◯ - Practicantes tradicionales (curanderos, yatiris, naturista, brujo…)
- ◯ - No hago ningún chequeo/control
- ◯ Otro (precisar)

5. ¿Ha escuchado hablar del Virus del Papiloma Humano (VPH)?

- ◯ - si
- ◯ - no

6. Si la respuesta a la pregunta anterior fue si, ¿cómo se ha usted informado?

- ◯ - He buscado sola la información
- ◯ - Me informé por medio de alguna persona cercana a mi
- ◯ - Me he informado por medio de los medios de comunicación
- ◯ - Me he informado por medio de mi médico generalista
- ◯ - Me he informado por medio de mi ginecólogo
- ◯ - Me informé por medio de una promotora

7. ¿Se ha realizado el test del Virus del Papilloma Humano (VPH)?

- ◯ - Si, lo hice
- ◯ - No, no lo hare nunca
- ◯ - Pienso hacer el despistaje muy pronto
- ◯ - No, me interesa hacerlo
- ◯ - No, pienso que eso no me concierne
- ◯ - No, el test me da miedo
- ◯ - No, conocer los resultados me daría miedo

8. ¿Piensa usted que hay algún tipo de problemas/dificultad para acceder al test de despistaje? (más de una opción son posibles)

- ☐ - Costo del test
- ☐ - Costo del transporte para acceder al lugar del test
- ☐ - Distancia para acceder al lugar del test
- ☐ - Actitud del personal de salud
- ☐ - El personal de salud no me explico bien
- ☐ - Mi marido/familia me impide hacer los test
- ☐ - El tiempo de espera de las citas
- ☐ - La dificultad de liberarme del trabajo
- ☐ - La dificultad de liberarme de mis obligaciones familiares
- ☐ - Ningún problema ha sido encontrado.

* 9. ¿ha hecho usted el test del Virus Papiloma Humano(VPH)?

- ◯ Si
- ◯ No

Si usted no ha hecho el test, no necesita responder a las siguientes preguntas

* 10. ¿Como se hizo el test de VPH?

- ○ - Autotoma
- ○ - Test realizado por el ginecólogo/personal de salud
- ○ - Test realizado durante el Papanicolau
- ○ - Yo no se
- ○ - Otro (precisar)

* 11. ¿Como recibió usted los resultados?

- ○ - Por llamada telefónica
- ○ - Por correo en mi casa
- ○ - Mediante el médico generalista
- ○ - Mediante mi ginecologo
- ○ - Otro (precisar)
- ○ - Via una promotora
- ○ - Via el laboratorio
- ○ - No he recibido ningún resultado del test

12. ¿Como le hubiera gustado a usted recibir los resultados? (más de una elección)

- ☐ - Por llamada telefónica
- ☐ - Por correo en mi casa
- ☐ - Mediante mi medico generalista
- ☐ - Otro (precisar)
- ☐ - Mediante mi ginecologo
- ☐ - Via una promotora
- ☐ - Via el laboratorio

13. Si usted tuviera resultados positivos, ¿ que preferiría usted?

- ○ - Hacer su seguimiento con un practicante tradicional (yatiri, curandero, naturista…)
- ○ - Hacer su seguimiento con el medico
- ○ - Hacer su seguimiento con el medico en colaboración con un practicante tradicional también
- ○ - Ninguno
- ○ - No lo se

* 14. ¿Cuál ha sido el resultado?

- - Positivo
- - Negativo
- - Aun no lo se/ No estoy segura
- - Un nuevo test debe ser realizado
- - No quiero dar mi resultado

Si su resultado ha sido negativo o que usted no recibió los resultados, no es necesario de responder a las siguientes preguntas

* 15. ¿Ha recibido usted los resultados del test de VPH o Papanicolau antes de la proposición de un seguimiento/tratamiento?

- ◯ - Si
- ◯ - Si, y No hago ningun seguimiento/tratamiento
- ◯ - No
- ◯ - No, y No hago ningun seguimiento/tratamiento
- ◯ - No quiero compartir esta informacion

* 16. ¿Dónde hace usted su seguimiento/tratamiento del VPH?

- ◯ - Hospital publico
- ◯ - Hospital privado
- ◯ - Centros de salud
- ◯ - Caja de salud
- ◯ - Médicos particulares o mi medico
- ◯ - Hago mi seguimiento con el ginecólogo del proyecto
- ◯ - Practicantes tradicionales (curanderos, yatiris, naturista, brujo…)
- ◯ - No hago ningun seguimiento/tratamiento
- ◯ Otro (precisar)

* 17. ¿Tuvo usted algún tipo de problemas para acceder al seguimiento/tratamiento? (más de una opción son posibles)

- ☐ - Costo del tratamiento/seguimiento
- ☐ - Costo del transporte para acceder al lugar del test
- ☐ - Distancia para acceder al lugar del test
- ☐ - Actitud del personal de salud
- ☐ - Explicación insuficiente del personal de salud
- ☐ - Mi marido/familia me impide hacer los test
- ☐ - El tiempo de espera de las citas
- ☐ - La dificultad de liberarme del trabajo
- ☐ - La dificultad de liberarme de mis obligaciones familiares
- ☐ - Ningún problema ha sido encontrado.
- ☐ - No quiero compartir esta informacion
- ☐ - Eso no me concierne

* 18. ¿Qué le dijo el médico después de darle los resultados positivos de VPH y la inspección?

- ○ - No hay ningún problema
- ○ - Usted tiene lesiones benignas
- ○ - Usted tiene lesiones graves
- ○ - Usted tiene un problema de infección
- ○ - Yo no se/ No estoy segura
- ○ - El doctor no me explico nada
- ○ - El doctor me explico pero no le entendi bien
- ○ - No quiero compartir esta informacion
- ○ - Necesita hacer otros examenes/control

19. ¿Qué tipo de seguimiento/tratamiento le han propuesto a usted?

- ○ - Histerectomía (retiramiento del útero o la matriz)
- ○ - Crioterapia/ Quemar la lesión
- ○ - Medicamentos
- ○ - Aun estoy esperando los resultados de los examenes
- ○ - Otro (precisar)
- ○ - No quiero compartir esta informacion
- ○ - Yo no se, el doctor no me explico bien
- ○ - Yo no se, el doctor me explico pero no entendi bien

* 20. ¿Recibió usted una explicación concerniendo el seguimiento/tratamiento del VPH?

- ○ - Si, y lo entendí
- ○ - Si, pero no entendí las explicaciones
- ○ - No
- ○ - No me interesa
- ○ - No quiero compartir esta informacion

* 21. ¿Está usted de acuerdo con lo que le propusieron?

- ○ - Sí, porque me lo explicaron todo
- ○ - Sí, porque hago confianza a mi medico
- ○ - Si pero no tengo los medios financieros de seguir mi tratamiento/mi terapia
- ○ - Sí, pero mi marido/mi familia no me permite de seguir mi tratamiento/mi terapia
- ○ - No, pero el médico debe tener razón/sabe lo que hace
- ○ - No, y no pretendo seguir lo que me han prescrito
- ○ - No se aun
- ○ - Eso no me concierne
- ○ - No quiero compartir esta informacion

Comentarios

22. ¿Tiene usted algunos comentarios sobre el seguimiento/tratamiento y/o el procedimiento de despistaje VPH?
