# Supplementary material for: Assessing Barriers Encountered by Women in Cervical Cancer Screening and Follow-Up Care in Urban Bolivia, Cochabamba
Source: Healthcare (Basel). 2022 Aug 24;10(9):1604. doi: 10.3390/healthcare10091604 (PMC9498362; doi:10.3390/healthcare10091604)
Supplement: Supplementary file 1 [file healthcare-10-01604-s001.zip › Supplementary Material File S4.pdf]

Cuestionario de investigación destinado al personal de salud

En el marco de un estudio realizado en cooperación entre la Universidad Libre de Bruselas y la Universidad Mayor de San Simon, estamos realizando un estudio para aumentar los conocimientos sobre las prácticas de seguimiento del Virus del Papiloma Humano, en la región de Cochabamba.
El objetivo de este estudio es, de comprender las posibles diferencias y las barreras que podría enfrentar el personal en el seguimiento de sus pacientes.

Todas las informaciones dadas en este cuestionario serán completamente anónimas, confidenciales y utilizadas únicamente en el marco de nuestra investigación universitaria.

1. ¿Donde trabaja usted? (más de una opción son posibles)

- ☐ - hospital publico
- ☐ - hospital privado
- ☐ - Centros de salud
- ☐ - Medicos particulares
- ☐ Otro(precisar)

2. ¿Es usted?

- ○ - Ginecólogo
- ○ - Generalista
- ○ - Enfermera
- ○ - Auxiliar de enfermera
- ○ - Bioquímico
- ○ - Otro (precisar)

3. ¿usted recibido una formación específica sobre la infección y detección del Virus del Papilloma Humano (VPH)?

- ○ - si
- ○ - no

4. ¿Informa usted a sus pacientes del riesgo de la infección por el VPH y su relación con el cáncer cervical (CC)?

- ○ - Si
- ○ - No

5. ¿Qué medio utiliza usted para despistar el VPH a sus pacientes? (más de una opción es posible)

- [ ] - Test de HPV
- [ ] - Papanicolaou
- [ ] - Inspección visual al ácido acético (IVAA)
- [ ] - Colposcopia
- [ ] - Biopsia
- [ ] - estudio histopatológico
- [ ] - Yo no estoy habilitada/habilitado para hacerlo
- [ ] Otro(precisar)

6. ¿Ha tenido usted algún problema/problemas para despistar a sus pacientes? Si si, cuales (más de una elección)

- [ ] - Costo del test
- [ ] - Falta de material para realizarlo
- [ ] - Yo no tengo la capacidad de hacerlo
- [ ] - Sobrecarga de trabajo
- [ ] - Falta de personal
- [ ] - Marido/familia de la paciente que impide la paciente de venir
- [ ] - Ninguna barrera ha sido encontrada
- [ ] Otro(precisar)

7. ¿Como anuncia usted los resultados del despistaje VPH a sus pacientes? (más de una elección)

- [ ] - Por llamada telefónica
- [ ] - Por correo
- [ ] - Via el médico generalista
- [ ] - Via el ginecólogo
- [ ] - Via una promotora
- [ ] - Espero que la paciente venga a verme
- [ ] - Le pido a un colega de hacerlo
- [ ] - Si la paciente utiliza la autotoma, ella vendrá hacia mi según los resultados
- [ ] Otro(precisar)

8. ¿Verifica usted los resultados antes de escoger el tipo de tratamiento para el VPH para la paciente?

- ○ - Si
- ○ - No
- ○ - Eso no me concierne

9. ¿A tenido usted problemas para realizar el seguimiento/ tratamiento de sus pacientes? Si si, cuáles? (más de una opción son posibles)

- ☐ - Costo del test
- ☐ - Falta de material
- ☐ - Falta de capacidad
- ☐ - Sobrecarga de trabajo
- ☐ - Falta de personal
- ☐ - Marido/familia de la paciente que impide las pacientes de venir
- ☐ - No, ningún problema ha sido encontrado
- ☐ Otro(precisar)

____

10. ¿Verifica usted a que estado de VPH la paciente se encuentra antes de entamar un tratamiento?

- ○ - Si, siempre
- ○ - Si, cuando es posible
- ○ - Non
- ○ - No pienso que eso sea necesario para tomar una decisión

11. ¿Da usted alguna explicación concerniente al seguimiento/tratamiento?

- ○ - Si
- ○ - Si, y verifico su nivel de comprensión
- ○ - No
- ○ - Mis colegas dan las explicaciones
- ○ - No se aplica a mi función

12. ¿Qué tipo de seguimiento/tratamiento que propone usted si las lesiones del cuello uterino son benignas? (más de una opción es posible)

- ☐ - Histerectomía
- ☐ - Crioterapia
- ☐ - Conización
- ☐ - Medicamentos
- ☐ - Ninguno
- ☐ Otro(precisar)

13. ¿Qué tipo de seguimiento/tratamiento propone usted si las lesiones son invasivas? (más de una opción es posible)

- ☐ - Histerectomía
- ☐ - Crioterapia
- ☐ - Conización
- ☐ - Medicamentos
- ☐ - Ninguno
- ☐ Otro(precisar)

14. ¿ Que tipo de seguimiento/ tratamiento propone usted si usted no está seguro de los resultados?

- ○ - - Histerectomía
- ○ - Crioterapia
- ○ - Conización
- ○ Otro(precisar)
- ○ - Medicamentos
- ○ - Ninguno

15. ¿Si la paciente no está de acuerdo con el seguimiento/ tratamiento propuesto, que le propone usted?

- ◯ - Reexplica para convencerla
- ◯ - Acepta su decisión y pide una nueva cita
- ◯ - Acepta su decisión, ella volverá si lo necesita
- ◯ - Acepta su decisión sin cuestionarse
- ◯ - Otro (precisar)
- ◯ - La transfiere a un otro profesional de salud
- ◯ - No le doy la opción de rechazar, es lo que hay que hacer
- ◯ - Nunca estuve frente a ese tipo de situación

16. ¿Piensa usted que las mujeres prefieren ir donde los practicantes tradicionales (yatiri, naturista, curandero…)?

- ◯ - Si
- ◯ - No
- ◯ - No se

17. ¿Estaría usted dispuesto a trabajar en colaboración con un practicante tradicional?

- ◯ - Si, actualmente ya lo hago
- ◯ - Si, si eso puede ayudar
- ◯ - No, prefiero que no
- ◯ - No, no encuentro eso útil
- ◯ - No lo se

18. ¿Trabajó usted con el proyecto VPH con la UMSS?

- ◯ - Si
- ◯ - no

19. ¿Tiene usted algunos comentarios sobre el seguimiento/tratamiento y /o el procedimiento de despistaje VPH?
